# Local and Landscape Effects on Carrion-Associated Rove Beetle (Coleoptera: Staphylinidae) Communities in German Forests

**DOI:** 10.3390/insects11120828

**Published:** 2020-11-24

**Authors:** Sandra Weithmann, Jonas Kuppler, Gregor Degasperi, Sandra Steiger, Manfred Ayasse, Christian von Hoermann

**Affiliations:** 1 Institute of Evolutionary Ecology and Conservation Genomics, University of Ulm, 89069 Ulm, Germany; jonas.kuppler@uni-ulm.de (J.K.); manfred.ayasse@uni-ulm.de (M.A.); 2 Richard-Wagnerstraße 9, 6020 Innsbruck, Austria; gregor.degasperi@gmail.com; 3 Department of Evolutionary Animal Ecology, University of Bayreuth, 95447 Bayreuth, Germany; Sandra.Steiger@uni-bayreuth.de; 4 Department of Conservation and Research, Bavarian Forest National Park, 94481 Grafenau, Germany; Christian.vonHoerman@npv-bw.bayern.de

**Keywords:** rove beetle communities, land use, forest management, carrion decomposition, piglet cadaver, forensic entomology

## Abstract

**Simple Summary:**

Increasing forest management practices by humans are threatening inherent insect biodiversity and thus important ecosystem services provided by them. One insect group which reacts sensitively to habitat changes are the rove beetles contributing to the maintenance of an undisturbed insect succession during decomposition by mainly hunting fly maggots. However, little is known about carrion-associated rove beetles due to poor taxonomic knowledge. In our study, we unveiled the human-induced and environmental drivers that modify rove beetle communities on vertebrate cadavers. At German forest sites selected by a gradient of management intensity, we contributed to the understanding of the rove beetle-mediated decomposition process. One main result is that an increasing human impact in forests changes rove beetle communities by promoting generalist and more open-habitat species coping with low structural heterogeneity, whereas species like *Philonthus decorus* get lost. Our results are not solely important for carrion ecological, but also for forensic entomological assessments on crime scenes, e.g., postmortem body relocation, because little information is available until now about rove beetles as one of the most important insect groups on bodies.

**Abstract:**

Intensification of anthropogenic land use is a major threat to biodiversity and thus to essential ecosystem services provided by insects. Rove beetles (Coleoptera: Staphylinidae), which react sensitively to habitat changes, are species-rich colonizers of vertebrate cadavers and contribute to the important ecosystem service of carrion decomposition. The unveiling of anthropogenic and environmental drivers that modify carrion-associated rove beetle communities should improve our understanding of the plasticity of cadaver decay. We report the presence of 80 rove beetle species on 65 decomposing piglet cadavers at forest sites characterized by a gradient of management intensity across three geographic regions in Germany. Local and landscape drivers were revealed that shape beetle abundance, diversity, and community composition. Forest management and regions affect rove beetle abundance, whereas diversity is influenced by local habitat parameters (soil pH, litter cover) and regions. The community composition of rove beetles changes with management intensification by promoting generalist species. Regarding single species, *Philonthus decorus* and *Anotylus mutator* are linked to unmanaged forests and *Ontholestes tessellatus* to highly used forest stands. The spatial information provided about carrion-associated rove beetle communities in German forests is not only of carrion-ecological but also of forensic entomological interest.

## 1. Introduction

The intensification and change of anthropogenic land use are amongst the main threats to inherent biodiversity [1,2]. In forests, the loss of biodiversity can be caused directly via the loss of population size because of harvest activities or the loss of microhabitats such as deadwood and litter and indirectly by the loss of the structural heterogeneity of habitat or prey [3,4]. Consequently, a decrease in species diversity in forests has been shown, for example, for various animal and plant taxa [5], aquatic arthropods in tree holes [4], terrestrial arthropods such as ambrosia beetles [6], copronecrophagous dung beetles [7], carrion-associated silphid beetles [8], and carabid and staphylinid beetles [3].

Rove beetles (Coleoptera: Staphylinidae) form one of the most diverse beetle families accounting for about 63,000 described species worldwide [9] and for about 1500 described species within German entomofauna. They inhabit almost every niche in terrestrial ecosystems [10,11]. In forests, some rove beetles have a high affinity for microhabitats such as litter or deadwood, but the majority of species are part of the forest soil and litter fauna [9,10,12]. Regarding deadwood inhabitants, there are certain staphylinid species like *Phloeonomus sjoebergi*, *Quedius plagiatus* and *Nudobius lentus* known that feed on bark beetles [13]. Rove beetles are also one of the most numerous colonizers of carrion [14,15,16]. On such ephemeral but highly dynamic resources, most rove beetles are predators that feed on Dipteran eggs and maggots at post-bloating and advanced decay stages or comprise species that are saprophagous (feeding on cadaveric fluids) and parasitize Dipteran pupae [17,18,19]. Hence, rove beetles are an important and species-rich insect group contributing to the essential ecosystem service of carrion decomposition. However, distinct soil and forest features are required for their occurrence on vertebrate cadavers and, hence, make rove beetles sensitive to habitat changes [10,20,21]. In other words, a change in environmental conditions can directly modify the “spatial” species pool of those rove beetles that are able to colonize vertebrate carrion and thus indirectly the decomposition process [20]. However, limited carrion-ecological work has been carried out on rove beetle communities found on carcasses [22,23], mainly because of poor taxonomic knowledge [15,16,24,25]. Therefore, a need exists for more in-depth data about rove beetle communities on vertebrate cadavers in the context of geographic regions and local habitat features [15,16,26].

In our study, we have investigated the effects of local and landscape factors on the colonization of 65 vertebrate cadavers by rove beetles in forests characterized by a gradient of management intensity. Specifically, we have raised the following questions:

1. Which environmental parameters determine carrion-associated rove beetle abundance, diversity, and community composition?
2. Which rove beetle species trapped on carrion are most affected by environmental drivers?

Our aim was to improve the general understanding of the ecosystem service provided by the way carrion-associated staphylinid beetles react to environmental parameters in differently managed forests at a large scale. Furthermore, we provide spatial information about rove beetle distributions in differently used forests and present a list of 80 rove beetle species found on small vertebrate cadavers by means of pitfall traps. Additionally, this study is a source of important basic-data knowledge for applied forensic entomological assessments since rove beetles are one of the most important groups in forensic entomological investigations [14,15]. These species have mainly been used for research on postmortem interval (PMI) estimations [22,27,28,29] or to indicate postmortem body relocation [15,16,30]; nevertheless, little information about this beetle group has been available until now.

## 2. Materials and Methods

### 2.1. Study Design

Our study is part of the “NecroPig” project within the framework of the interdisciplinary research project Biodiversity Exploratories (DFG Priority Program 1374, www.biodiversity-exploratories.de, [31]). We conducted fieldwork in three distinct study regions of Germany, namely Schwäbische Alb (ALB) in the southwest, Hainich-Dün (HAI) in the middle, and Schorfheide-Chorin (SCH) in the northeast. Within the Biodiversity Exploratories, experimental forest sites were selected to cover the whole gradient of forest management intensity. This gradient was quantified via a precalculated index, named the silvicultural management intensity (SMI) index implemented by [32], which describes the intensity of land use of a forest stand based on main tree species, stand age, and amount of living and dead wooden biomass. The value of the SMI index ranges between 0 for unmanaged stands up to 1 for intensively managed stands. Consequently, experimental forest sites ranged from near-natural forests (unmanaged and extensively managed beech forests with low SMI indices) to intensively managed monocultures (selection system with beech stands, age class forests with beech, pine or spruce forests with high SMI indices) representing the spectrum of actual silvicultural management in German forests. For a more detailed description of the “Biodiversity Exploratories” study design, forest site descriptions and further the implementation of the silvicultural management intensity index, see [31,32].

### 2.2. Exposure of Piglet Cadavers

**Figure 1:**
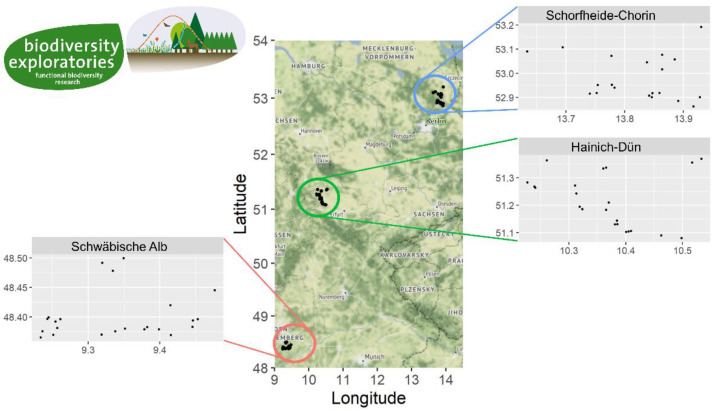
Geographic positions (x-axis: longitude, y-axis: latitude) of 75 experimental study sites within three study regions of the Biodiversity Exploratories project in Germany. Blue—Schorfheide-Chorin (SCH) region, green—Hainich-Dün (HAI) region and red—Schwäbische Alb (ALB) region.

In August 2014, we exposed 75 stillborn piglet cadavers (1.4 kg average weight) simultaneously at each of 75 experimental forest sites across the three study regions in Germany (Figure 1). Piglets were placed inside wire cages (63 cm × 48 cm × 54 cm, MH Handel GmbH, Munich, Germany) to prevent vertebrate scavenging. After exposure on day 0, we visited the cadavers at seven subsequent sampling events: on days 2, 4, 6, 9, 16, 23, and 30. At each sampling event, we used a standardized protocol to sample, for example, bacteria, soil, insects, and carrion scent. A detailed overview of the standardized sampling procedure is given in [8]. We lost data from 10 piglets during the exposure times because of temporary federal prohibitions and the complete scavenging of piglet cadavers by foxes that dug holes to enter the cages. Therefore, data from 65 sites were included in our calculations.

### 2.3. Insect Sampling

Lured insects were captured using two pitfall traps, one in front of the head and another at the anus of each piglet cadaver. The traps consisted of biodegradable plastic cups (500 mL) that were filled with a water/scent-free detergent solution and covered with plastic rain shields. The traps were opened for a standardized 48 h period before each sampling event. During sampling, trapped insects were transferred into plastic containers filled with 70% ethanol for preservation. We identified all trapped rove beetles to the species level based on [18,33,34,35,36].

### 2.4. Environmental Variables

We installed data loggers (Thermochron iButton, Whitewater, WI, USA) inside each wire cage to record ambient air temperature every 30 min over the whole decomposition period. Information concerning further abiotic and biotic characteristics of the experimental forest sites was obtained from the Biodiversity Exploratories data platform “BExIS” (Biodiversity Exploratories Information System, https://www.bexis.uni-jena.de). We selected environmental parameters representing forest characteristics that have been suggested to affect the diversity of rove beetles [9,10,11,37,38,39]. These selected explanatory variables are as follows: region, silvicultural management intensity index (SMI), mineral soil pH, soil temperature (°C), silt content (g/kg soil), understory proportion, Shannon index of vascular plants, litter cover (%), deadwood cover (% ), ambient air temperature (°C), elevation (m), soil moisture (%), and soil type. Detailed information about the environmental variables included in the analyses is shown in Table S1.

### 2.5. Statistical Analyses

Generalized linear models (GLMs), redundancy analysis (RDA), and rank abundance curves were performed in R (version 3.6.2) [40]. All samples that were collected over seven sampling events at one experimental forest site were pooled to calculate the total abundance of lured rove beetles at one carcass. Rove beetle diversity (species richness and Shannon diversity) was calculated for each forest site by using the functions “specnumber” and “diversity” (package “vegan”, [41]). We employed two mathematical measurements to evaluate species diversity in order to obtain a complete picture of diversity variation: species richness is sensitive to rare species, whereas Shannon diversity is equally sensitive to rare and abundant taxa [42].

#### 2.5.1. Data Exploration

For data exploration, we followed the protocol of [43]. We checked for outliers within the dataset by using dot plots. All models that we calculated in an initial approach were clearly dependent on one outlier (forest site in ALB region (AEW3) with 491 rove beetle individuals). To ensure that our result was robust and not driven by one extreme outlier, we excluded the respective experimental forest site from our calculations. Next, collinearity within the initial explanatory environmental variables was checked by using pair plots with Pearson’s correlation coefficients (PCC; function “cor” in the “stats” package, [40] and variance inflation factors (GVIF; function “corvif”, [44]). Therefore, one of two explanatory variables that resulted in high correlations (PCC > (+/−) 0.75 and GVIF > 4) were excluded, namely ambient air temperature, soil moisture, and elevation. Furthermore, soil type as a categorial variable was also excluded due to the high correlation to the three study regions [31]. All final continuous explanatory variables were then transformed using the function ((x − mean(x))/sd(x)) to normalize data mean values to 0 and standard deviations to 1. After removal of variables contributing to high correlation, the initial model had the following specification, based on the final set of explanatory variables: “response variable” ~ ”SMI” + “pH” + “understory” + “soil temperature” + “Shannon index for plants” + “litter cover” + “deadwood cover” + “silt content” + “region”. We treated “region” as an additional covariate instead of a random effect to be able to analyze its influence.

#### 2.5.2. Effects of Forest Management, Habitat Parameters, and Region on Rove Beetle Abundance and Diversity

In order to analyze the effects of forest management, environmental parameters and region on the response variables “abundance” and “species richness”, we fitted negative binomial generalized linear models (GLM) by using the function “glm.nb” in the “MASS” package [45]. For the response variable “Shannon index”, we fitted a log-linked Gaussian GLM with the function “glm” (“stats” package, [40]). For each response variable, we dropped each explanatory variable step-by-step (using “stepAIC”, package “MASS”, [45]) and selected the best fitted model based on Akaike´s information criterion (AIC). We accepted the model with the lowest AIC as the final model. After model selections, the final GLM models were specified as:

“abundance” ~ ”SMI” + “pH” + “understory” + “shannon plants” + “region”;

“richness” ~ ”SMI” + “pH” + “shannon plants” + “litter cover” + “deadwood cover” + “region”;

“shannon” ~ ”SMI” + “pH” + “shannon plants” + “litter cover” + “silt content” + “region”.

To test for effects of the covariates included in the model, we used analysis of deviance (F-test) implemented in the “ANOVA” function (package “car”, [46]). With regard to the categorial variable region, we performed a posthoc test for correction of pairwise comparisons (function “emmeans”, package “emmeans”, [47]). After each GLM analysis, we simulated model residuals and confirmed that model assumptions, e.g., no overdispersion, zero inflation, and spatial autocorrelation, were met to validate the model fit (package “DHARMa”, [48]). Marginal effects of multiple regression analyses were visualized by using the package “sjPlot” [49].

#### 2.5.3. Effects of Forest Management, Habitat Parameters, and Region on Rove Beetle Community Composition

In order to assess the influence of forest management, habitat parameters, and region on rove beetle community compositions, we performed a redundancy analysis (RDA; function “rda” in the package “vegan”, [41,50]). This ordination technique is a direct gradient analysis and relates the observed beetle community matrix, or the response variables, to a corresponding matrix of environmental variables, or the explanatory variables. Prior to analysis, the response matrix was Hellinger-transformed via the “decostand” function as recommended by [51] to generate a correlation matrix with low weights being given to rare species; explanatory variables were identically standardized as in GLMs. We used the same initial model specification as in GLMs to allow comparisons. Using the function “ordistep” we stepwise-reduced variables from the initial model to select the model that best explained the variation in beetle community data. As a result, the final RDA model was specified as: “species matrix” ~ ”region” + SMI” + “pH”. Variance inflation factors (VIF) < 3 (“vif.cca”, package “vegan”, [41]) indicated no high collinearity within the environmental predictors. Statistical significance of the final model was determined by applying the “ANOVA” function (package “car”, [46]). We used abundance instead of presence/absence data because the information of abundance is an important determinant of community structures [3]. We then visualized RDA results in a 2D correlation triplot (“ordiplot” function, type 2 scaling with weighted sums of species scores, package “vegan”, [41]). In such a correlation plot, the focus is on response correlations, and the cosine of angles between all vectors reflects the correlations [50]. Additionally, vector distances from the center point were considered for interpretation.

#### 2.5.4. Effects of Environmental Variables on Single Rove Beetle Species

To check for the specific proportional contribution of single rove beetle species to the dissimilarity between the groups of the most influential environmental parameters previously evaluated in the RDA analysis (which were “region” and “SMI”), we performed one-way ANOSIM (analysis of similarities) and post hoc SIMPER (similarity percentage) analysis by using PRIMER 6 (version 6.1.15, [52]). SIMPER analysis calculates the proportional contribution of each species to the similarity within and dissimilarity between groups based on a Bray–Curtis dissimilarity matrix. R values specified the level of similarity (R = 0: no difference between groups, R = 1: larger similarities within the group than between the groups). Since SMI index values were continuous data, we had previously grouped them evenly into three categories named “low” (0.002–0.2), “medium” (0.21–0.4), and” high” (0.41–0.6) to be able to use this variable in the SIMPER analysis.

#### 2.5.5. Rank Abundance Curves

Rank abundance curves for displaying relative species abundance of all collected rove beetles on piglet cadavers at 65 forest sites were built in R by using the functions “rankabundance” and “rankabunplot” (package “BiodiversityR”, [53]).

## 3. Results

### 3.1. Rove Beetles on Piglet Cadavers

In total, 2692 adult rove beetle individuals belonging to 8 subfamilies, 30 genera, and 80 species were collected on 65 piglet cadavers at the various experimental forest sites in Germany (N = 65 sites). The most abundant specimen numbers (≥100 individuals) were found for nine species: *Tachinus pallipes* Gravenhorst (404 ind.), *Bisnius fimetarius* Gravenhorst (338 ind.), *Philonthus addendus* Sharp (310 ind.), *Anotylus mutator* Lohse (206 ind.), *Philonthus succicola* Thomson (185 ind.), *Tachinus laticollis* Gravenhorst (166 ind.), *Philonthus tenuicornis* Mulsant and Rey (124 ind.), *Ontholestes tessellatus* Geoffroy (123 ind.) and *Philonthus splendens* Fabricius (109 ind.). Twenty species (<100 and ≥10 ind.) were considered as subdominant taxa, and 51 remaining species (<10 ind.) were rare taxa (Table S2).

### 3.2. Effects of Forest Management, Habitat Parameters, and Region on Rove Beetle Abundance and Diversity

Forest management (SMI) and the three study regions explained the variation in rove beetle abundance best. Beetle abundance significantly increased in forests with increasing anthropogenic management intensity (Table 1, Figure S1a). Furthermore, higher numbers of rove beetles were found on experimental forest sites where mineral soil pH was higher (Figure S1b). However, in forests where the understory proportion was higher, we captured significantly fewer rove beetles (Table 1, Figure S1c). A significant influence of regions was noted on rove beetle abundance (Table 1, Figure S1d). More rove beetles were trapped in the SCH region than in either of the other two regions ALB and HAI (Table 1). The Shannon diversity of vascular forest plants did not affect rove beetle abundance on piglet cadavers (Table 1).

The strongest effects on rove beetle diversity (evaluated by species richness and Shannon diversity) were exhibited by the parameters of mineral soil pH, litter cover, and study regions (Table 1). Rove beetle diversity increased with higher mineral soil pH and forest litter cover (Figures S2a,b and S3a,b). Furthermore, a significant impact of study regions was detected on beetle diversity (Figures S2c and S3c). The highest rove beetle diversity was found in the SCH region, followed by the ALB and HAI regions. Anthropogenic forest management intensity did not affect rove beetle diversity. Moreover, the Shannon diversity of vascular forest plants, deadwood cover, and silt content did not affect rove beetle diversity on piglet cadavers.

### 3.3. Effects of Forest Management, Habitat Parameters, and Region on Rove Beetle Community Composition

**Figure 2:**
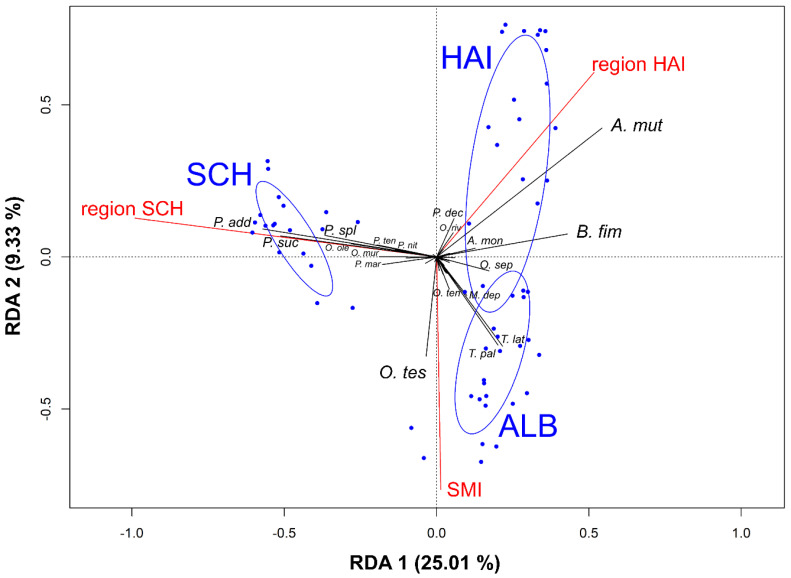
Correlation triplot of the first two axes obtained from the redundancy analysis (RDA) showing the relationship between rove beetle communities on carrion and important environmental parameters (sites as blue points and blue ellipses for region groups, species scores as black lines and labels and explanatory variables as red lines and labels). Only significant explanatory variables are shown. To provide better visualization, arrowheads for vectors were not included, and only 19 important species regarding their importance from a total of 80 species were plotted (see Table S2 for species abbreviation). ALB = Schwäbische Alb, HAI = Hainich-Dün, SCH = Schorfheide-Chorin, SMI = silvicultural management intensity index; *A. mut* = *A. mutator*, *B. fim* = *B. fimetarius*, *O. tes* = *O. tessellatus*, see Table S2 for all species abbreviations.

Two environmental variables, namely forest management (SMI) and region, were the most important variables influencing staphylinid community variation (ANOVA_SMI_: df = 1, F = 5.545, *p* < 0.001, ANOVA_region_: df = 2, F = 9.755, *p* < 0.001, Figure 2). Moreover, RDA showed that environmental variables explained 38% (34.34% for adjusted R²) of the variation in rove beetle community data, with axis 1 and axis 2 explaining 25.01% and 9.33% of the variance, respectively. The first two to three canonical axes together explained 34.33–36.99% of the variation between rove beetle species and 90.5–97.5% of the species-environment relationship (Table S3). Region SCH was the most important variable for the separation on the first axis, and rove beetle communities within region SCH were clearly distinct from beetle communities within regions ALB and HAI. Forest management (SMI) contributed most to the explained variance on the second axis. Region HAI was important for the separation on the third axis.

### 3.4. Effects of Environmental Variables on Single Rove Beetle Species

As the study regions and forest management (SMI) explained most of the variance in rove beetle communities revealed by RDA analysis (see above), we further examined which single rove beetle species were mostly affected. RDA showed that three *Philonthus* species were more correlated to region SCH (*P. addendus, P. succicola*, *P. splendens*), whereas two *Tachinus* species (*T. pallipes* and *T. laticollis)* were stronger linked to region ALB (Figure 2). Moreover, *A. mutator* and *B. fimetarius* were associated with region Hainich-Dün, and a higher number of *O. tessellatus* beetles was collected where forest management was more intensive (higher SMI values). *Philonthus decorus* Gravenhorst was the most negatively correlated species with the SMI axis (lower SMI values).

**Figure 3:**
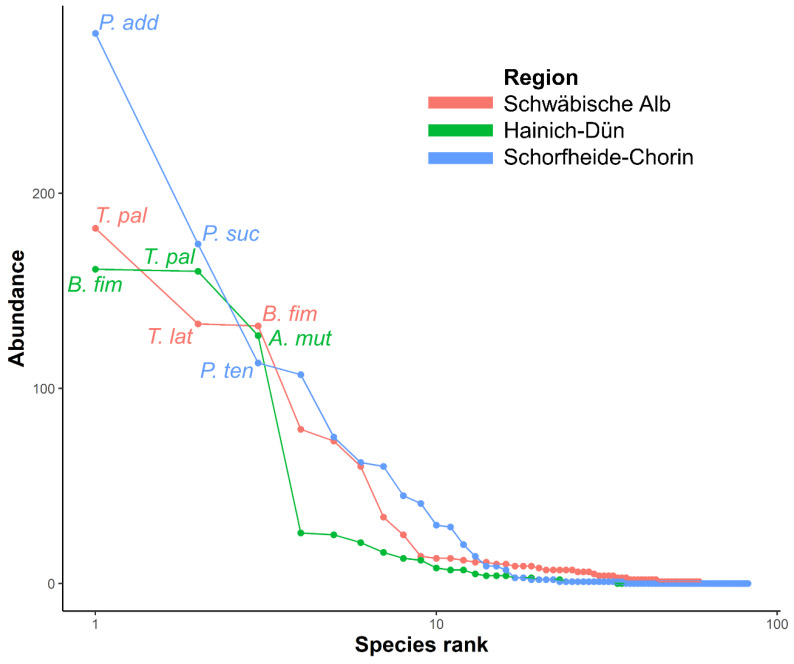
Rank abundance curves showing the abundance of rove beetle species observed on piglet cadavers at 65 forest sites separated for each study region: Schwäbische Alb, Hainich-Dün and Schorfheide-Chorin. The three most abundant species are labeled for each study region (*P. add* = *P. addendus*, *P. suc* = *P. succicola*, *P. ten* = *P. tenuicornis*, *T. pal* = *T. pallipes*, *T. lat* = *T. laticollis*, *B. fim* = *B. fimetarius*, *A. mut* = *A. mutator*), N = 2692 individuals.

Additionally, on inspection of the single influence of the study regions, ANOSIM analysis revealed that rove beetle community composition on piglet carrion differed significantly between all three regions (Table S4 and depicted in Figure 3). The most abundant species collected on piglet cadavers in region ALB were *T. pallipes*, *B. fimetarius* and *T. laticollis*. These three species contributed at almost 58% to the similarity within this region (SIMPER, Table S5). Similarly, *B. fimetarius*, *T. pallipes* and *A. mutator* were collected in the highest amounts in region HAI. These species had a cumulative contribution of 84% to the similarity within this region. In contrast, *P. addendus*, *P. succicola*, and *P. tenuicornis* made up the three most abundant species in region SCH. Together with *P. splendens*, they contributed more than 79% to the similarity within this region. Remarkably, over 280 individuals of *P. addendus* were captured on piglet cadavers in this study region. Detailed information of those species that contributed to the similarity within and dissimilarity between regions is given in Tables S5 and S6.

With regard to the single effect of forest management intensity on rove beetle composition found on carrion, the ANOSIM results showed that community composition between management levels “low”, “medium”, and “high” differed significantly (Table S4). *Anotylus mutator*, *B. fimetarius* and *P. decorus* were the main taxa describing the communities in forests with low management intensity and contributed cumulatively with almost 73% to the similarity within these forests (SIMPER, Table S7). Nine species explained 92.39% of the community composition at low forest management intensity, and interestingly, the single species *A. mutator* accounted for more than 40% within the low SMI levels. Forests with medium management intensity were characterized by *T. pallipes*, *B. fimetarius*, and *P. addendus* with a cumulative contribution of about 50%. Eleven species explained 90.2% of the community composition at medium management intensity. However, only four species, namely *T. pallipes*, *B. fimetarius*, *O. tessellatus*, and *T. laticollis*, were typical for forests with a high management level. These four species explained 92.6% of the community composition for highly used forests. Remarkably, *O. tessellatus* alone accounted for 19.7% within high management levels compared with only 5.5% in medium used forests and 2.6% in low used forests. *Bisnius fimetarius* was well presented in all groups of SMI levels and accounted for coequally (16.62–23.7%) to all groups. Furthermore, *A. mutator* and *P. decorus*, which were part of the three main taxa mainly contributing to forests with low SMI levels, were less abundant in forests with contrasting SMI levels.

Rove beetle communities differed by 81.82% between forests with medium and high management intensity. Rove beetle composition also differed by 81.71% on average between forests with medium and low management intensity, but the largest difference was found in rove beetle communities between high and low SMI levels with an average dissimilarity of 86.41% (complete information about SIMPER dissimilarities between different management levels can be found in Table S8).

## 4. Discussion

Our study contributes to a general understanding of the environmentally driven dynamics of the rove beetle community and its consequences for their provided ecosystem service on decomposing vertebrate biomass. We identified 80 rove beetle species that were lured to piglet cadavers at distinct forest sites in Germany. *Tachinus pallipes*, *B. fimetarius*, and *P. addendus*, which comprised about 39% of the total catch, were the most dominant species found on carrion. Our large-scale study revealed environmental characteristics at local and landscape scales, and that represent important drivers shaping rove beetle abundance, diversity, and community composition on vertebrate cadavers. Forest management positively affected rove beetle abundance by changing the community compositions but had no influence on beetle diversity (species richness and Shannon diversity). Instead, local habitat parameters such as soil pH and litter cover shaped beetle diversity. Apart from these findings, a clear effect of the geographic locations of the Biodiversity Exploratories on overall rove beetle abundance, diversity and community composition was seen at the landscape scale. In particular, the abundance and occurrence of some *Philonthus* species (namely *P. addendus, P. succicola*, and *P. splendens*) were clearly correlated to the study region SCH, *A. mutator* and *B. fimetarius* to the HAI region, and *T. pallipes* and *T. laticollis* to the ALB region. Furthermore, *O. tessellatus* was associated with highly managed forests and *P. decorus* and *A. mutator* with unmanaged forest stands.

### 4.1. Local and Landscape Parameters Affecting Rove Beetle Abundance, Diversity, and Communities on Vertebrate Carrion

The overall abundance of rove beetles found on carrion increased significantly with increasing forest management and decreasing understory proportion, whereas community compositions changed. The ecological dynamics regarding anthropogenic management in forests seem to be different from those in grasslands that have shown a clear decrease of arthropod abundance, e.g., grasshoppers, because of intensive mowing and grazing [54]. Tree gaps, induced by tree felling as one common management practice, may favor a few abundant species, including invasive and pest species, to an even higher extent [55] and can promote more open-habitat species than closed-canopy species [3,56]. Indeed, we were able to show that some abundant beetle species, namely *T. pallipes*, *B. fimetarius*, *O. tessellatus*, and *T. laticollis*, have a positive correlation with increasing forest management. In particular, *O. tessellatus* may benefit from increasing forest management, an idea supported by its large distance from the ordination centroid (Figure 2), and can therefore be interpreted as a “winner species” coping with management intensification in forests. *Ontholestes tessellatus* is described as a generalist predaceous species ([18], Table S2) that can deal with low structural habitat heterogeneity, and that has an affinity to open habitats [16]. Since *A. mutator* and *P. decorus* do not occur in large numbers at intensively managed forest sites, these species can be interpreted as particularly vulnerable to management intensification (“loser species”). In the study of [16], *P. decorus* was the only species exclusively found on carrion in forests in a comparison of grassland and forest sites in Poland. Thus, *P. decorus* exhibits a preference for forest habitats, more precisely unmanaged forests and can therefore be considered as a potential indicator species for near-natural forest stands. Hence, our results suggest that extensive forest utilization can change the community composition of carrion-associated rove beetles (with potential consequences for the overall decomposition process) by mainly supporting and establishing generalist and open-habitat species [57].

However, we found that forest management did not affect rove beetle diversity. Instead, higher litter cover on the forest floor increased the diversity of carrion-associated rove beetles. This finding contrasts with that of [3], who have reported higher species richness of ground-dwelling staphylinid beetles in managed forest stands. Nevertheless, since densely forested and shady coniferous monocultures at medium to high management levels are characterized by higher litter cover (abscission of needles) and low understory vegetation (lack of light), forest management is therefore indirectly linked to litter cover and understory proportion. Most rove beetles living in the forest soil and litter fauna require distinct microhabitats [58], and increasing litter cover makes more microhabitats available and boosts habitat heterogeneity at small scales, which in turn may promote a higher rove beetle diversity.

Since our three study regions differ in climate, soil conditions, and tree species [31], the influence of the region on beetle diversity and abundance was to be expected. Schorfheide-Chorin, as the northernmost, largest, and most sparsely populated region, showed a clear divergent pattern. Staphylinid abundance and diversity were highest in this region, and rove beetle community composition was clearly distinct from the other two regions, namely HAI and ALB. Most likely, this regional influence can be explained by the unique post-glacial sandy soils (better suited for beetle penetration and dwelling) covered with large pine and oak forests [31] that make the SCH region such an outstanding study region. In accordance with the present study, other animal-related investigations within the Biodiversity Exploratories have also shown that the SCH region is inhabited by vast arthropod assemblages, e.g., copronecrophagous dung beetle and carrion beetle masses on exposed vertebrate carrion [7,8] and others [3,59,60,61,62]. Von Hoermann [7] interpreted massive assemblages of dung beetles on carrion in the SCH region with the existence of large populations of game animals (red deer *Cervus elaphus*) exclusively in this region of the Biodiversity Exploratories [63]. This interpretation may also be applicable to our results in rove beetles since greater numbers of large vertebrates may increase the occurrence of large cadavers in these forests and thus the food provisioning for large-sized predatory rove beetles (e.g., *Philonthus* species, Table S2) by huge maggot masses at these resource pulses. Hence, a diverse and highly abundant staphylinid community could have been established in this region over time.

Regarding single rove beetle species, our study clearly shows that the three study regions explain most of the constrained variance (Figure 2) in rove beetle community composition. Rove beetles of the genus *Philonthus* (*P. addendus*, *P. succicola*, *P. splendens*) were more abundant in the SCH region and *A. mutator* and *B. fimetarius* in the HAI region, whereas two *Tachinus* species (*T. pallipes*, *T. laticollis*) were more abundant in the ALB region. *Philonthus succicola* (former name is *P. chalceus* Stephens) and *P. addendus* are described as abundant species across Germany, most notably in northern, plain, and flat areas [64], which is one of the main characteristics of the SCH region [31]. Furthermore, all the above-mentioned *Philonthus* species (*P. addendus*, *P. succicola*, *P. splendens*, and *B. fimetarius* as a synonym for *Philonthus fimetarius* Gravenhorst) were detected in high numbers on adult pig cadavers in studies in Western Poland [16,29] in three different forests. In particular, *Philonthus splendens* was linked to hornbeam-oak forests, which resemble typical, managed forests in the SCH region (pine-oak forests with sandy soils and low understory vegetation with almost the same latitude as the SCH region in Germany). However, both *Tachinus* species and *A. mutator* were not detected [16] or were only trapped at low abundance [15,29] on large cadavers in Western Poland and Belgium, suggesting a potential preference for small vertebrate cadavers; further research on small piglet cadavers must be performed to validate this suggestion. With regard to their geographical distribution, *T. pallipes* and *T. laticollis* show a preference for hilly and mountainous areas [65], as can also be found in the ALB region, which is the hilliest among the three investigated regions [31]. Early references for the distribution of *A. mutator* lack because its taxonomic separation from *A. sculpturatus* Gravenhorst did not occur until 1963 [66]. However, *A. sculpturatus* has been described in the former literature as being a species more abundant in southern and western Germany [66], connecting this species with our study region ALB in which it (*A. mutator*) is the fifth most contributing species to the average similarity of the rove beetle community composition within this region.

The overall effect of higher mineral soil pH values on higher rove beetle abundance and diversity cannot directly be explained by our outstanding study region SCH as mentioned above, since the soils in the SCH region are characterized by the lowest pH values compared with the other two study regions. We conclude that another variable in the model explains the high abundance of rove beetles in the SCH region. However, in accordance with our findings across all three study regions, [38] have reported soil pH as one of the most important factors controlling the diversity of staphylinid beetles. The total abundance, the total number of hygrophilous rove beetle species, and the number of species and individuals dependent on decaying substrates increase with increasing soil pH values [38].

### 4.2. Impact on Forensic Entomological Assessments

When studying the decomposition of animal biomass in the context of basic and applied forensic entomology, the biology of key insect groups such as flies (Calliphoridae, Sarcophagidae, Muscidae) and beetles (Silphidae, Staphylinidae, Dermestidae) is of great importance [14,28,67,68]. The spatial distribution patterns of the relevant insect groups and the environmental effects on their diversity and community composition, as found on cadavers, provide valuable information for spatial crime-scene assignments and can improve the quality of forensic conclusions in legal investigations, such as whether bodies have been moved postmortem between different habitats [16,30]. We have shown that some rove beetle species have geographic preferences, such as *Philonthus* species for the SCH region, *A. mutator* and *B. fimetarius* for the HAI region, and *T. pallipes* and *T. laticollis* for the ALB region in Germany. Such regional-specific results may be important for reliable PMI estimations on-site. Furthermore, *P. decorus* and *A. mutator* may be indicator species of body relocations from unmanaged forest stands, and *O. tessellatus* may be an indicator species for human corpse infestations in highly managed forest stands. Thus, the new insights provided here could be important not only for carrion-ecological but also for forensic entomological applications. On this point, further studies are needed that investigate how specific are the species for the carrion habitat (obligate versus facultative necrophilic) supporting our conclusions for forensic significance.

## 5. Conclusions

The intensification of anthropogenic forest management is a major threat for inherent biodiversity and thus for the essential ecosystem services provided by insects, such as the decomposition of vertebrate biomass. The Staphylinidae are one of the most diverse beetle groups on Earth, are sensitive to land-use changes, and are important colonizers of decomposing animal remains. The unveiling of the anthropogenic and environmental drivers that modify the pool of rove beetle species on vertebrate carrion may improve our understanding of the plasticity of this important ecosystem service. Our results indicate that only a few species show distinct local and landscape responses to aspects such as geographic regions and forest management practices and that increasing forest management changes the community composition of carrion-associated rove beetles by promoting generalist and more open-habitat species such as *O. tessellatus*.

## Figures and Tables

**Table 1:** Results of the best fitted generalized linear model (GLM) analyses showing the effects of forest management (SMI index), environmental parameters, and region on rove beetle abundance and diversity (species richness, Shannon diversity) on piglet cadavers at 65 forest study sites. ED (significant effect direction) ↑/↓ = positive/negative, F = F value, *p* = significance level. Significant (*p* < 0.05) *p*-values are given in bold. Intercepts are not shown. Post hoc tests for estimated marginal means are shown for the categorial variable region.

**Negative Binomial GLM: Response Variable “Abundance”**				
**Covariates**	**Estimate (ED)**	**Standard Error**	**F**	***p***
SMI index	0.446 (↑)	0.128	14.85	**<0.001**
Mineral soil pH	0.271 (↑)	0.135	5.20	**0.026**
Understory proportion	−0.449 (↓)	0.155	8.94	**0.004**
Shannon plants	0.225	0.14	2.86	0.096
Region			12.92	**<0.001**
	ALB–HAI: 0.0622 ALB–SCH: −1.7871 ↓ HAI–SCH: −1.8493 ↓	ALB–HAI: 0.241 ALB–SCH: 0.443 HAI–SCH: 0.393		ALB–HAI: 0.964 ALB–SCH: **<0.001** HAI–SCH: **<0.001**
**Negative Binomial GLM: Response Variable “Species Richness”**				
**Covariates**	**Estimate (ED)**	**Standard Error**	**F**	***p***
SMI index	0.127	0.078	2.32	0.133
Mineral soil pH	0.33 (↑)	0.075	17.21	**<0.001**
Shannon plants	0.136	0.083	2.45	0.123
Litter cover	0.257 (↑)	0.082	8.61	**0.005**
Deadwood cover	0.1	0.065	2.06	0.157
Region	ALB–HAI: 0.478 ↑ ALB–SCH: −0.641 ↓ HAI–SCH: −1.119 ↓	ALB–HAI: 0.124 ALB–SCH: 0.222 HAI–SCH: 0.224	14.98	**<0.001** ALB–HAI: **<0.001** ALB–SCH: **0.011** HAI–SCH: **<0.001**
**Gaussian GLM: Response Variable “Shannon Diversity”**				
**Covariates**	**Estimate (ED)**	**Standard Error**	**F**	***p***
SMI index	0.092	0.049	3.43	0.069
Mineral soil pH	0.171 (↑)	0.052	11.55	**0.001**
Shannon plants	0.089	0.053	2.86	0.097
Litter cover	0.219 (↑)	0.057	15.7	**<0.001**
Silt content	−0.129	0.071	3.13	0.082
Region	ALB–HAI: 0.309 ↑ ALB–SCH: −0.1 HAI–SCH: −0.408	ALB–HAI: 0.091 ALB–SCH: 0.197 HAI–SCH: 0.208	6.71	**0.002** ALB–HAI: **0.002** ALB–SCH: 0.868 HAI–SCH: 0.121

ALB = Schwäbische Alb, HAI = Hainich-Dün, SCH = Schorfheide-Chorin.

## Supplementary Materials

The following are available online at https://www.mdpi.com/2075-4450/11/12/828/s1, Figure S1 (A–D): Marginal effects of multiple regression between the total abundance of rove beetles on piglet cadavers at 65 forest sites and (A) forest management intensity, (B) mineral soil pH, (C) forest understory proportion, and (D) region. For (A–C) regression lines and 95% confidence intervals for negative binomial GLMs are shown. For (D) box plots show the median and interquartile range for negative binomial GLMs. Different letters indicate significant differences among groups with estimated marginal means post hoc tests (*p* < 0.05). ALB = Schwäbische Alb, HAI = Hainich-Dün, SCH = Schorfheide-Chorin, Figure S2 (A–C): Marginal effects of multiple regression between species richness of rove beetles on piglet cadavers at 65 forest sites and (A) mineral soil pH, (B) forest litter cover, and (C) region. For (A,B) regression lines and 95% confidence intervals for negative binomial GLMs are shown. For (C) box plots show the median and interquartile range for negative binomial GLMs. Different letters indicate significant differences among groups with estimated marginal means post hoc tests (*p* < 0.05). ALB = Schwäbische Alb, HAI = Hainich-Dün, SCH = Schorfheide-Chorin, Figure S3 (A–C): Marginal effects of multiple regression between Shannon diversity of rove beetles on piglet cadavers at 65 forest sites and (A) mineral soil pH, (B) forest litter cover, and (C) region. For (A,B) regression lines and 95% confidence intervals for Gaussian GLMs are shown. For (C) box plots show the median and interquartile range for Gaussian GLMs. Different letters indicate significant differences among groups with estimated marginal means post hoc tests (*p* < 0.05). ALB = Schwäbische Alb, HAI = Hainich-Dün, SCH = Schorfheide-Chorin, Table S1: Environmental variables included in the analyses, undertaken on the BExIS platform (Biodiversity Exploratories Information System, https://www.bexis.uni-jena.de), Table S2: List of species of all collected rove beetles on decomposing piglet cadavers at 65 forest study sites in three distinct study regions in descending order by total abundance. Additional information for each species is given for subfamily, size class, ecological niche, and food preference [1,2,3,4,5,6,7,8,9], Table S3: Summary of statistical results of the redundancy analysis (RDA). Significant (*p* < 0.05) values are given in bold, Table S4: Results of pairwise ANOSIM analyses showing the effects of region and SMI on the community composition of rove beetles on piglet cadavers at 65 forest study sites. Significant (*p* < 0.05) *p*-values are given in bold, Table S5: Results of SIMPER analysis showing those species that contributed most to the similarity of the rove beetle community composition within regions: average abundance (%) of characteristic species in each region (ALB, HAI, SCH), their contribution to the within-group similarity (%), and the cumulative total (%) of contributions (90% cutoff), Table S6: Results of SIMPER analysis showing those species that contributed most to the dissimilarity of the rove beetle community composition between regions: average abundance (%) of characteristic species for each region (ALB, HAI, SCH), their contribution to the between-group dissimilarity (%), and the cumulative total (%) of contributions (90% cutoff), Table S7: Results of SIMPER analysis showing those species that contributed most to the similarity of the rove beetle community composition within silvicultural management intensity (SMI) levels: average abundance (%) of characteristic species at each SMI level (LOW, MEDIUM, HIGH), their contribution to the within-group similarity (%), and the cumulative total (%) of contributions (90% cutoff), Table S8: Results of SIMPER analysis showing those species that contributed most to the dissimilarity of the rove beetle community composition between silvicultural management intensity (SMI) levels: average abundance (%) of characteristic species for each SMI level (LOW, MEDIUM, HIGH), their contribution to the between-group dissimilarity (%), and the cumulative total (%) of contributions (90% cutoff).
