# Supplementary material for: Local and Landscape Effects on Carrion-Associated Rove Beetle (Coleoptera: Staphylinidae) Communities in German Forests
Source: Insects. 2020 Nov 24;11(12):828. doi: 10.3390/insects11120828 (PMC7760899; doi:10.3390/insects11120828)
Supplement: Supplementary file 1 [file insects-11-00828-s001.pdf]

Supplementary Materials

Local and Landscape Effects on Carrion-Associated Rove Beetle (Coleoptera: Staphylinidae) Communities in German Forests

Sandra Weithmann[1*], Jonas Kuppler[1], Gregor Degasperi[2], Sandra Steiger[3], Manfred Ayasse[1], Christian von Hoermann[4]

[1] Institute of Evolutionary Ecology and Conservation Genomics, University of Ulm, 89069 Ulm, Germany; jonas.kuppler@uni-ulm.de (J.K.), manfred.ayasse@uni-ulm.de (M.A.)
[2] Richard-Wagnerstraße 9, 6020 Innsbruck, Austria; gregor.degasperi@gmail.com (G.D.)
[3] Department of Evolutionary Animal Ecology, University of Bayreuth, 95447 Bayreuth, Germany; Sandra.Steiger@uni-bayreuth.de (S.S.)
[4] Department of Conservation and Research, Bavarian Forest National Park, 94481 Grafenau, Germany; Christian.vonHoerman@npv-bw.bayern.de (C.v.H.)
* Correspondence: sandra.weithmann@uni-ulm.de (S.W.)

The supplementary material is structured in sections Materials and methods and Results.

Materials and methods

Table S1: Environmental variables included in the analyses undertaken on the BExIS platform (Biodiversity Exploratories Information System, https://www.bexis.uni-jena.de).

Variable	Variable type	Categories or description of variable	unit	Data source information
region	categorial	Schwäbische Alb (ALB), Hainich-Dün (HAI), Schorfheide-Chorin (SCH)	-	ID: 20826, version: 1.14.10, owner: Nieschulze, Schulze, Fischer, Ayasse, Weisser, Ostrowski, König-Ries
SMI index	continuous	silvicultural management intensity (SMI) index (from 0 to 1)<br>0 = undisturbed<br>1 = disturbed	-	ID: 17746, version: 1.2.2; owner: Schall & Ammer
mineral soil pH	continuous	pH-measurement in the mineral forest soil<br>(0 - 10 cm)	-	ID: 19067, version: 4.1.2; owner: Schöning, Klötzing, Schäfer, Gan, Schrumpf, Trumbore
soil temperature	continuous	forest soil temperature at 10 centimeters below surface	°C	ID: 19007, version: 1.0.5; owner: Wöllauer, Hänsel, Nauss, Forteva
silt content	continuous	amount of medium silt (particle size 0.0063 - 0.02 mm) in forest soil samples	g/kg soil	ID: 14686, version: 1.9.6; owner: Schöning, Solly, Klötzing, Trumbore, Schrumpf
understory proportion	continuous	forest understory proportion up to 2 m above the ground	-	ID: 17066, version: 1.1.3; owner: Schall & Ammer
Shannon plants	continuous	Shannon diversity index for all vascular plants in forests (based on vegetation relevés on all 400 m^2 forest sites by visual estimation)	-	ID: 16806, version: 1.2.2; owner: Grassein & Fischer
litter cover	continuous	forest litter cover in summer	%	ID: 6240, version: 1.6.16; owner: Boch, Socher, Mueller, Prati, Fischer
deadwood cover	continuous	forest deadwood cover	%	ID: 6240, version: 1.6.16; owner: Boch, Socher, Mueller, Prati, Fischer

Results

Table S2: List of species of all collected rove beetles on decomposing piglet cadavers at 65 forest study sites in three distinct study regions in descending order by total abundance. Additional information for each species is given for subfamily, size class, ecological niche, and food preference [1–9].

Species name (author, year)	Subfamily	Size class	Ecological niche	Food	Total abundance	Abundance in region 'ALB'	Abundance in region 'HAI'	Abundance in region 'SCH'	Abbreviation for analyzes
Tachinus pallipes (Gravenhorst, 1806)	Tachyporinae	III	de, sap	z	404	182	160	62	T_pal
Bisnius fimetarius (Gravenhorst, 1802)	Staphylininae	III	de, cp, fu	z	338	132	161	45	B_fim
Philonthus addendus (Sharp, 1867)	Staphylininae	IV	de	z	310	12	16	282	P_add
Anotylus mutator (Lohse, 1963)	Oxytelinae	II	de, cp	sa (?)	206	79	127	0	A_mut
Philonthus succicola (Thomson, 1860)	Staphylininae	V	de, cp	z	185	7	4	174	P_suc
Tachinus laticollis (Gravenhorst, 1802)	Tachyporinae	II	de, sap	z	166	133	26	7	T_lat
Philonthus tenuicornis (Mulsant & Rey, 1853)	Staphylininae	IV	de, cp	z	124	6	5	113	P_ten
Ontholestes tessellatus (Geoffroy, 1785)	Staphylininae	V	de, cp, ca	z	123	73	21	29	O_tes
Philonthus splendens (Fabricius, 1793)	Staphylininae	V	de, cp, sap	z	109	2	0	107	P_spl
Philonthus decorus (Gravenhorst, 1802)	Staphylininae	V	de, hyg, mu	z	91	25	25	41	P_dec
Philonthus marginatus (Müller, 1764)	Staphylininae	IV	de, cp	z	88	13	0	75	P_mar
Megarthrus depressus (Paykull, 1789)	Proteininae	I	de, cp, hu, fu	sa, m (?)	64	60	2	2	M_dep

Ontholestes murinus (Linné, 1758)	Staphylininae	V	de, ca	z	61	1	0	60	O_mur
Omalium septentrionis (Thomson, 1857)	Omaliinae	II	de, hu, fu	z (?)	41	34	7	0	O_sep
Ocypus olens (Müller, 1764)	Staphylininae	V	hyg, hu	z	30	0	0	30	O_ole
Atheta monticola (Thomson, 1852)	Aleocharinae	I	de, sap	z (?)	27	14	12	1	A_mon
Philonthus nitidus (Fabricius, 1787)	Staphylininae	V	de, cp	z	20	0	0	20	P_nit
Quedius cinctus (Paykull, 1790)	Staphylininae	IV	de, cp	z	15	13	2	0	Q_cin
Atheta dadopora (Thomson, 1867)	Aleocharinae	I	myc, st, de	z (?)	15	11	1	3	A_dad
Aleochara curtula (Goeze, 1777)	Aleocharinae	II-IV	de, cp	pa, z	14	4	1	9	A_cur
Atheta crassicornis (Fabricius, 1793)	Aleocharinae	II	de, sap	z (?)	14	6	7	1	A_cra
Lordithon lunulatus (Linné, 1760)	Tachyporinae	III	myc, fu	z	14	0	13	1	L_lun
Staphylinus erythropterus (Linné, 1758)	Staphylininae	V	hyg, hu	z	14	0	0	14	S_ery
Atheta castanoptera (Mannerheim, 1830)	Aleocharinae	II	de, myc, fu	z (?)	13	9	4	0	A_cas
Megarthrus stercorarius (Mulsant & Rey, 1878)	Proteininae	I	de, cp, fu	sa, m (?)	11	11	0	0	M_ste
Philonthus varians (Paykull, 1789)	Staphylininae	III	de, cp	z	11	1	1	9	P_var
Atheta fungi (Gravenhorst, 1806)	Aleocharinae	I	de, hu	z	10	10	0	0	A_fun.1

Ocypus tenebricosus (Gravenhorst, 1846)	Staphylininae	V	hyg, hu	z	10	10	0	0	O_ten
Atheta hansseni (Strand, 1943)	Aleocharinae	I	de, fu	z (?)	10	7	3	0	A_han
Atheta britanniae (Bernhauer & Scheerpeltz, 1926)	Aleocharinae	I	de, myc	z (?)	9	9	0	0	A_bri
Proteinus brachypterus (Fabricius, 1792)	Proteininae	I	de, myc, hu	sa, m (?)	9	9	0	0	P_bra
Atheta sodalis (Erichson, 1837)	Aleocharinae	I	de, myc, fu	z (?)	8	7	1	0	A_sod
Megarthrus nitidulus (Kraatz, 1857)	Proteininae	I	de, cp, fu	sa, m (?)	8	8	0	0	M_nit
Omalium rivulare (Paykull, 1789)	Omaliinae	II	de, sap, fu	sa	8	0	8	0	O_riv
Quedius lateralis (Gravenhorst, 1806)	Staphylininae	V	de, sap, su, fu	z	8	6	1	1	Q_lat
Atheta cinnamoptera (Thomson, 1856)	Aleocharinae	I	de, cp	z (?)	7	4	3	0	A_cin
Quedius lucidulus (Erichson, 1839)	Staphylininae	III	de, hu	z	7	7	0	0	Q_luc
Tachinus proximus (Kraatz, 1855)	Tachyporinae	III/IV	de, cp	z	7	7	0	0	T_pro
Atheta laevana (Mulsant & Rey, 1852)	Aleocharinae	I	de, cp, si	z (?)	6	4	2	0	A_lae
Anotylus sculpturatus (Gravenhorst, 1806)	Oxytelinae	II	de, cp	sa	5	3	2	0	A_scu
Atheta picipes (Thomson, 1856)	Aleocharinae	I	de, fu, co	z (?)	5	4	0	1	A_pic
Pella cognata (Märkel, 1842)	Aleocharinae	III	myr, hu	sa (?)	5	5	0	0	P_cog

Atheta intermedia (Thomson, 1852)	Aleocharinae	II	de, cp	z (?)	4	0	4	0	A_int
Rugilus rufipes (Germar, 1836)	Paederinae	III	de, hyg	z	4	1	1	2	R_ruf
Atheta boreella (Brundin, 1948)	Aleocharinae	I	de, hyg	z (?)	4	2	0	2	A_bor
Omalium rugatum (Mulsant & Rey, 1880)	Omaliinae	II	de	z (?)	3	3	0	0	O_rug
Philonthus politus (Linné, 1758)	Staphylininae	IV/V	de, ca	sa (?)	3	0	0	3	P_pol
Platystethus arenarius (Geoffroy, 1785)	Oxytelinae	II/III	de, cp	z	3	3	0	0	P_are
Quedius mesomelinus skoraszewskyi (Korge, 1960)	Staphylininae	IV	de, ni	z	3	0	3	0	Q_mes
Aleochara stichai (Likovsky, 1965)	Aleocharinae	II	de	pa, z	2	2	0	0	A_sti
Atheta ravilla (Erichson, 1839)	Aleocharinae	I	de, fu, ni	z (?)	2	2	0	0	A_rav
Ocalea picata (Stephens, 1832)	Aleocharinae	II/III	de, hyg, mu	z (?)	2	2	0	0	O_pic
Othius subuliformis (Stephens, 1833)	Staphylininae	III	de, hu	sa, z (?)	2	2	0	0	O_sub
Placusa tachyporoides (Waltl, 1838)	Aleocharinae	I	co	m	2	2	0	0	P_tac
Xantholinus laevigatus (Jacobsen, 1849)	Staphylininae	IV	de, hyg	z	2	2	0	0	X_lae
Bisnius pseudoparcus (Brunne, 1976)	Staphylininae	I	de	z	2	0	0	2	B_pse

Acrotona parvula (Mannerheim, 1830)	Aleocharinae	I	de, cp	z (?)	1	0	0	1	A_par
Aleochara funebris (Wollaston, 1864)	Aleocharinae	II	de	pa, z	1	1	0	0	A_fun
Aleochara intricata (Mannerheim, 1830)	Aleocharinae	II/III	de, sap	pa, z	1	0	0	1	A_intr
Anotylus inustus (Gravenhorst, 1806)	Oxytelinae	II	de, cp, xer	sa, z (?)	1	1	0	0	A_inu
Anthobium atrocephalum (Gyllenhal, 1827)	Omaliinae	II	de, hu, fu	sa, z (?)	1	1	0	0	A_atr
Atheta gagatina (Baudi di Selve, 1848)	Aleocharinae	I	de, myc, fu	z (?)	1	0	0	1	A_gag
Atheta paracrassicornis (Brundin, 1954)	Aleocharinae	I	de, myc	z (?)	1	1	0	0	A_par.1
Autalia longicornis (Scheerpeltz, 1947)	Aleocharinae	I	de, myc	z (?)	1	1	0	0	A_lon
Geostiba circellaris (Gravenhorst, 1806)	Aleocharinae	I	hyg, hu	z (?)	1	1	0	0	G_cir
Lathrobium brunnipes (Fabricius, 1793)	Paederinae	IV	de, hyg	z	1	0	0	1	L_bru
Megarthrus denticollis (Beck, 1817)	Proteininae	I	de, cp, hu	sa, m (?)	1	1	0	0	M_den
Megarthrus prosseni (Schatzmayr, 1904)	Proteininae	I	de, cp, fu	sa, m (?)	1	0	0	1	M_pro
Ocypus brunnipes (Fabricius, 1781)	Staphylininae	V	de, hu, hyg	z	1	0	0	1	O_bru
Oxypoda brevicornis (Stephens, 1832)	Aleocharinae	I/II	z (?)	z	1	1	0	0	O_bre
Oxytelus laqueatus (Marsham, 1802)	Oxytelinae	II/III	de, cp	sa	1	0	1	0	O_laq

Pella lugens (Gravenhorst, 1802)	Aleocharinae	II	myr	sa	1	1	0	0	P_lug
Philonthus laminatus (Creutzer, 1799)	Staphylininae	IV	de, sap	z	1	0	0	1	P_lam
Quedius fuliginosus (Gravenhorst, 1802)	Staphylininae	V	de, hu, hyg	z	1	0	0	1	Q_ful
Rugilus mixtus (Lohse, 1956)	Paederinae	III	de	z	1	1	0	0	R_mix
Tachinus rufipes (Linné, 1758)	Tachyporinae	III	de, sap	z	1	1	0	0	T_ruf
Bolitochara tecta (Assing, 2014)	Aleocharinae	III	myc, si, pp, bo, xde	z	1	0	1	0	B_tec
Gyrohypnus punctulatus (Paykull, 1789)	Staphylininae	III/IV	de	z	1	0	1	0	G_pun
Pella humeralis (Gravenhorst, 1806)	Aleocharinae	III	myr, hu	sa, z (?)	1	1	0	0	P_hum
Plataraea brunnea (Fabricius, 1798)	Aleocharinae	II	de, hu, xer	z	1	0	1	0	P_bru

ALB = Schwäbische Alb, HAI = Hainich-Dün, SCH = Schorfheide-Chorin

Key for 'size class': I= 1 - 3 mm, II= 3 - 4.5 mm, III= 4.5 - 7 mm, IV= 7 - 11 mm, V= > 11 mm

Key for 'ecological niche' (and explanation [lives on or in]): bo= boleticolous (pored fungi), ca= cadavericolous (carrion), cp= coprophilic (feces), co= corticolous (bark), de= detriticolous (detritus), fu= fungicolous (fungi), hu= humicolous (humus), hyg= hygrophilic (humidity), mu= muscicolous (moss), myc= mycetophilic (mushrooms), myr= myrmecophilic (ants), ni= nidicolous (bird-nest), pp=polyporicolous (sponges), sap= saprophilic (decomposing material), si= silvicolous (forests), st= stercolicolous (dung), su= succicolous (plant sap), xer= xerophilic (drought), xde= xylodetriticolous (woody detritus)

Key for 'food' (and explanation [feeds on]): z= zoophagous (animal material), s= saprophagous (decomposing material), p= parasitoid (lives as parasitoid), (?)= unpublished information (G.D.) based on literature [1-9] of near-related species.

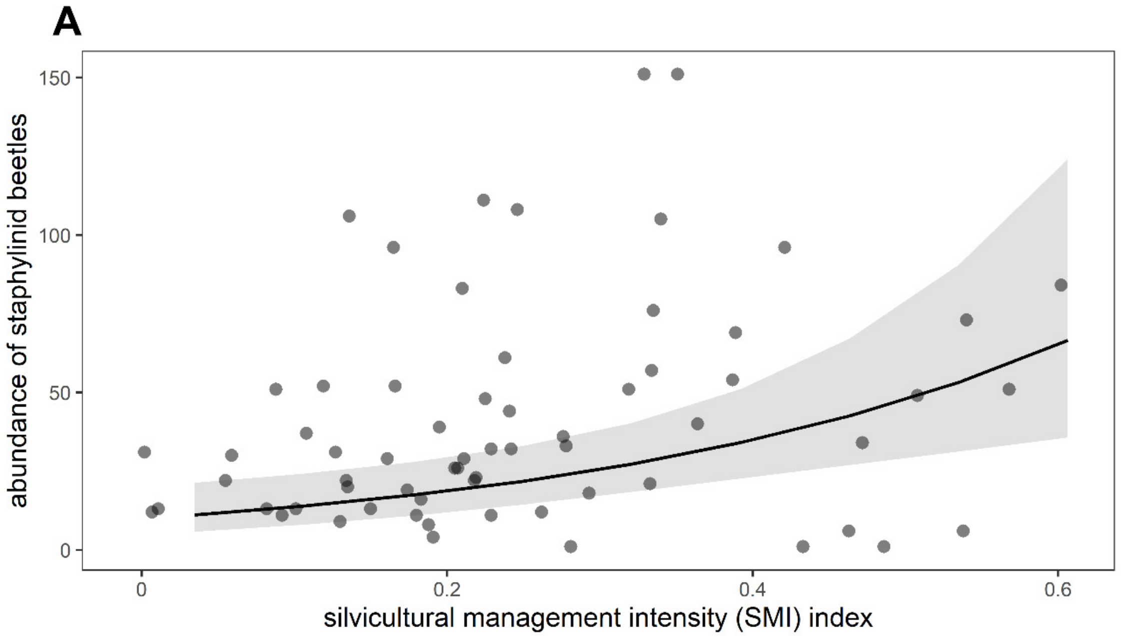
A
abundance of staphylinid beetles
150
100
50
0
0
0.2
0.4
0.6
silvicultural management intensity (SMI) index

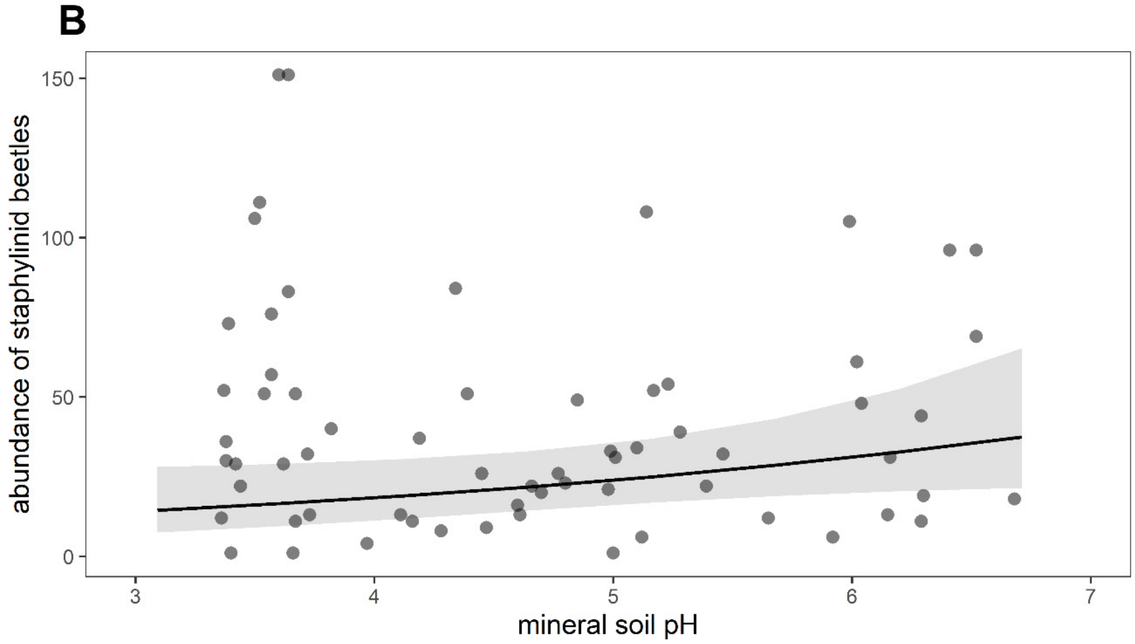
B
abundance of staphylinid beetles
150
100
50
0
3
4
5
6
7
mineral soil pH

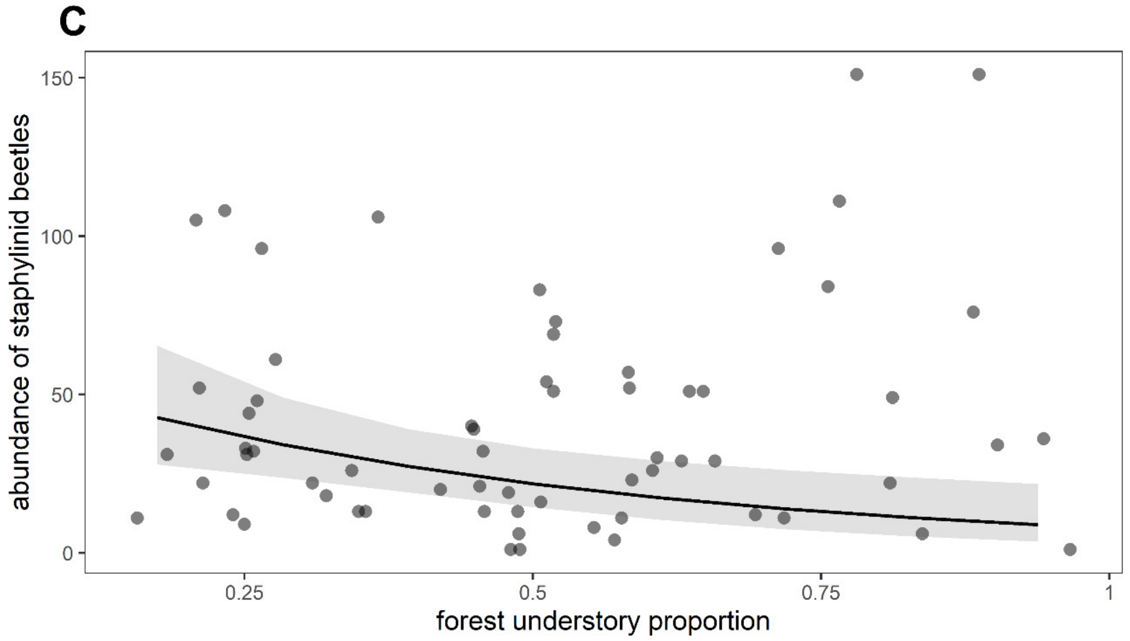

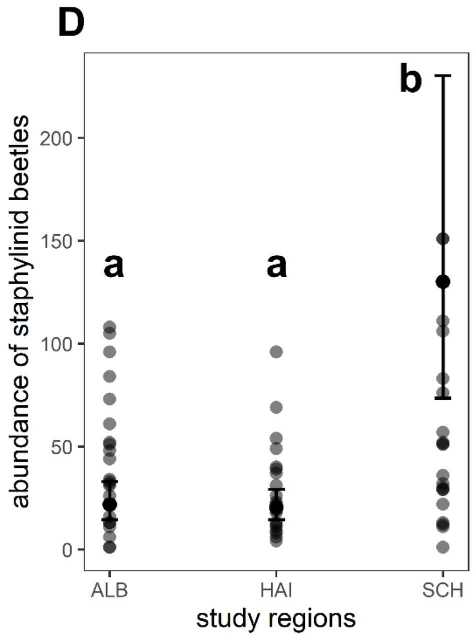

Figure S1 A – D: Marginal effects of multiple regression between total abundance of rove beetles on piglet cadavers at 65 forest sites and A) forest management intensity, B) mineral soil pH, C) forest understory proportion, and D) region. For A - C) regression lines and 95% confidence intervals for negative binomial GLMs are shown. For D) box plots show the median and interquartile range for negative binomial GLMs. Different letters indicate significant differences among groups with estimated marginal means post-hoc tests (p < 0.05). ALB = Schwäbische Alb, HAI = Hainich-Dün, SCH = Schorfheide-Chorin.

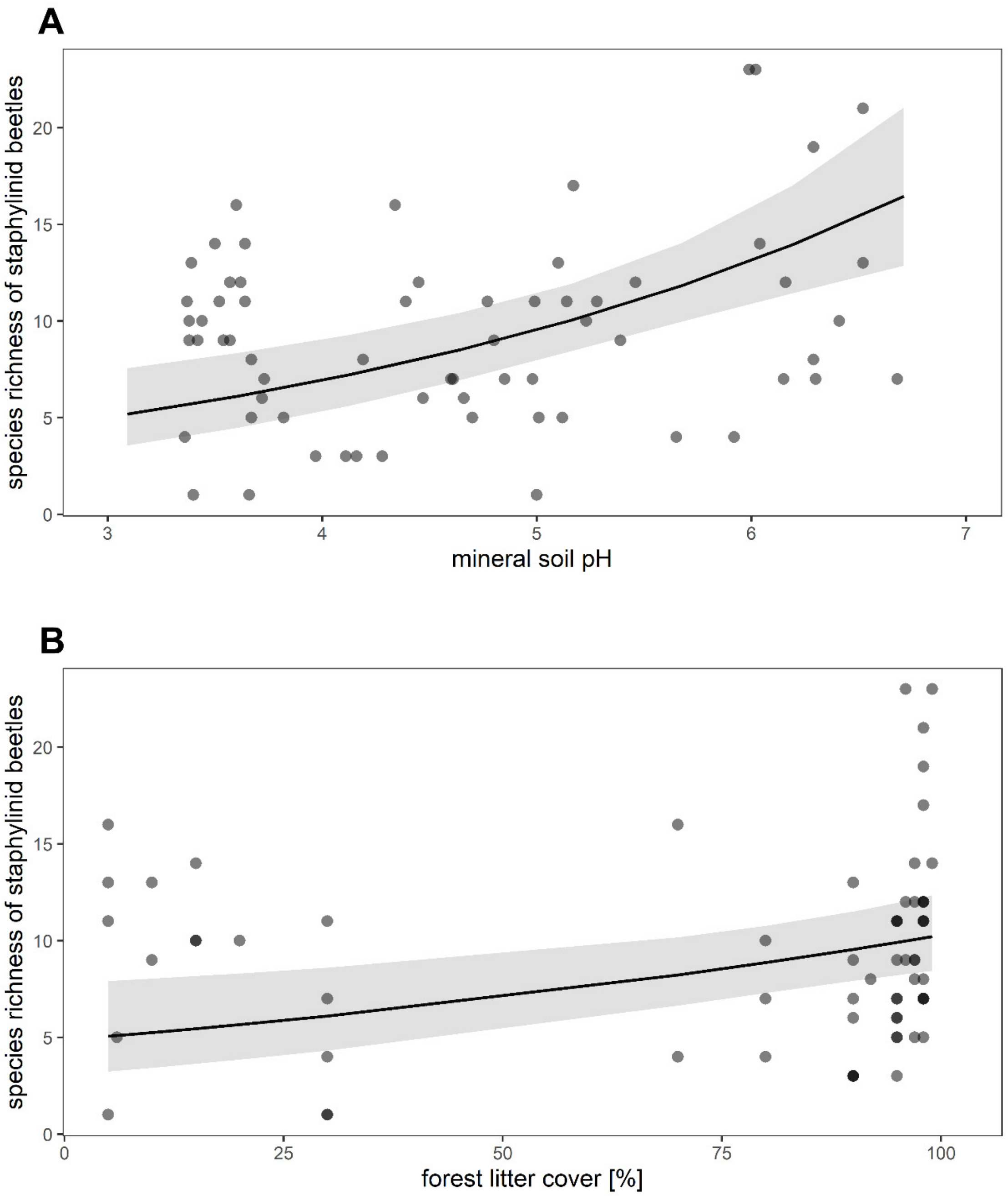
A
species richness of staphylinid beetles
mineral soil pH
B
species richness of staphylinid beetles
forest litter cover [%]

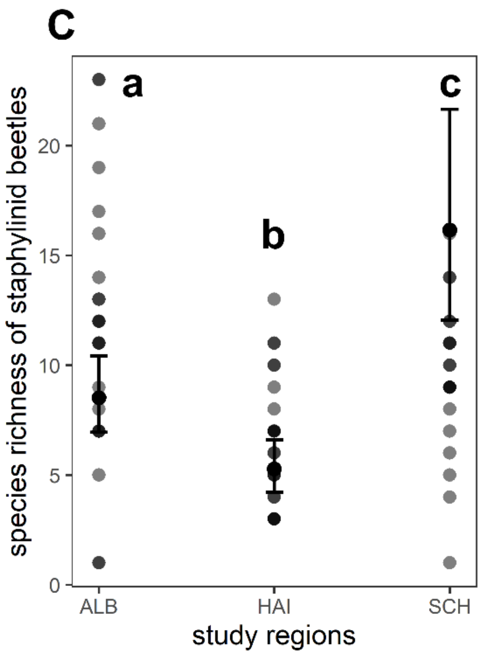

Figure S2 A – C: Marginal effects of multiple regression between species richness of rove beetles on piglet cadavers at 65 forest sites and A) mineral soil pH, B) forest litter cover, and C) region. For A - B) regression lines and 95% confidence intervals for negative binomial GLMs are shown. For C) box plots show the median and interquartile range for negative binomial GLMs. Different letters indicate significant differences among groups with estimated marginal means post-hoc tests ($p < 0.05$). ALB = Schwäbische Alb, HAI = Hainich-Dün, SCH = Schorfheide-Chorin.

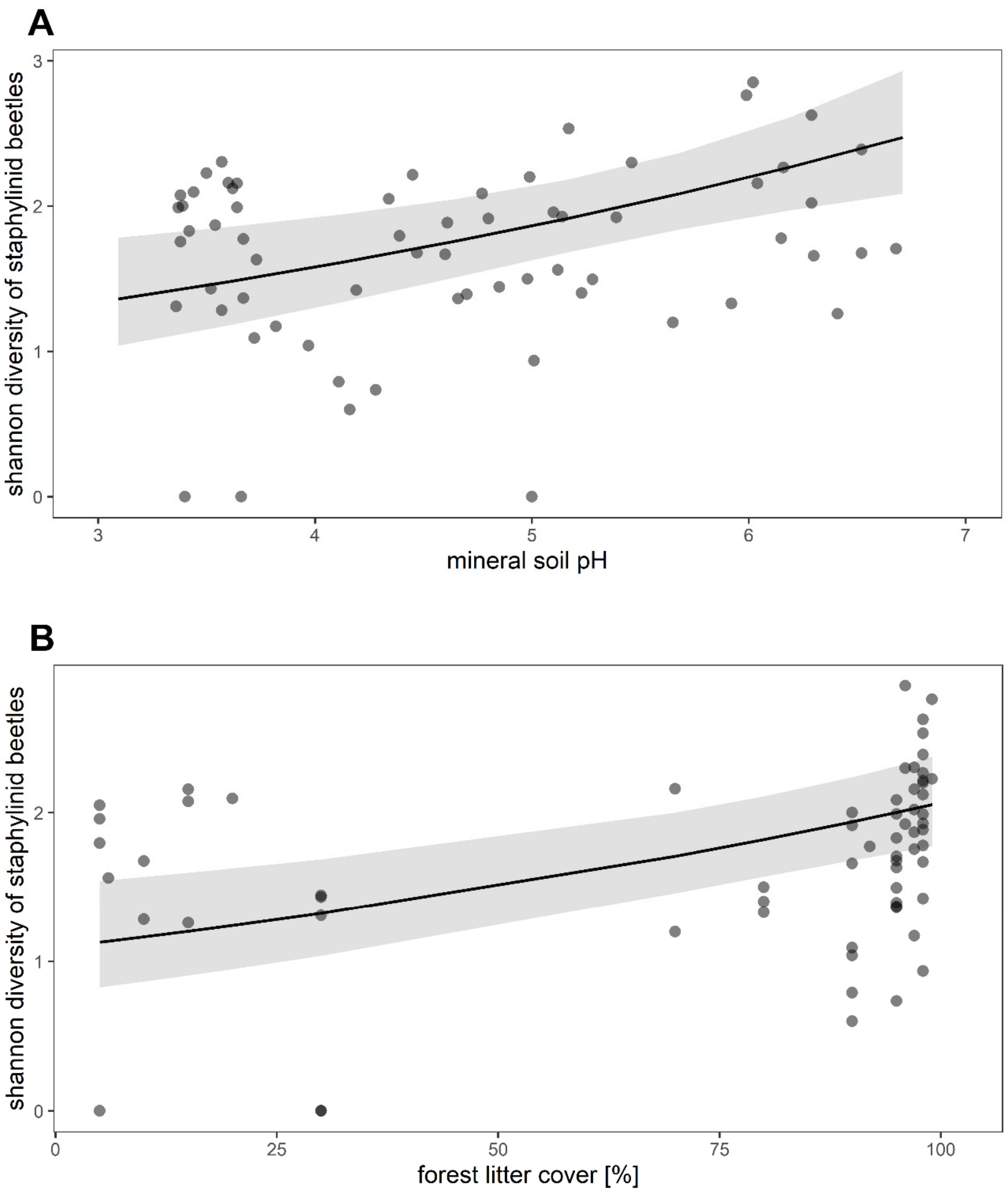
A
shannon diversity of staphylinid beetles
mineral soil pH
0
1
2
3
3
4
5
6
7
B
shannon diversity of staphylinid beetles
forest litter cover [%]
0
1
2
0
25
50
75
100

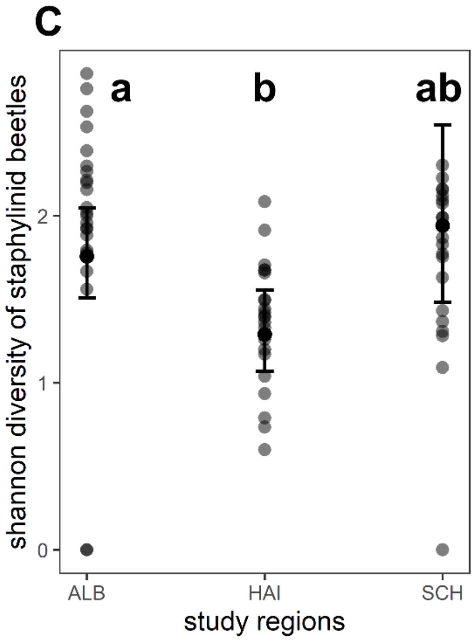

Figure S3 A – C: Marginal effects of multiple regression between Shannon diversity of rove beetles on piglet cadavers at 65 forest sites and A) mineral soil pH, B) forest litter cover, and C) region. For A - B) regression lines and 95% confidence intervals for Gaussian GLMs are shown. For C) box plots show the median and interquartile range for Gaussian GLMs. Different letters indicate significant differences among groups with estimated marginal means post-hoc tests ($p < 0.05$). ALB = Schwäbische Alb, HAI = Hainich-Dün, SCH = Schorfheide-Chorin.

Table S3: Summary of statistical results of the redundancy analysis (RDA). Significant ($p < 0.05$) values are given in bold.

**Partitioning of variance**			
	Total variance		Proportion
Total	0.645		1.000
Constrained	0.245		0.38
Unconstrained	0.4		0.621
	Canonical axes		
	**Axis 1**	**Axis 2**	**Axis 3**
Eigenvalue	0.161	0.06	0.017
Percentage explained	25.01	9.33	2.66
**Cumulative percentage variance**			
for species	25.01	34.33	36.99
for species-environment relation	65.9	90.5	97.5
**ANOVA** (forward tests for axes)			
Variance	0.161	0.06	0.017
F value	24.178	9.017	2.571
p value	**0.001**	**0.001**	**0.006**
**Correlations**			
region HAI	0.536	0.571	**-0.613**
region SCH	**-0.984**	0.16	0.061
SMI	0.003	**-0.74**	-0.584
**Coefficients**			
region HAI	0.011	0.174	**-0.248**
region SCH	**-0.291**	0.027	-0.184
SMI	-0.019	**-0.083**	-0.097

p = significance level, SMI = silvicultural management intensity index,
ALB = Schwäbische Alb, HAI = Hainich-Dün, SCH = Schorfheide-Chorin.

Table S4: Results of pairwise ANOSIM analyses showing the effects of region and SMI on the community composition of rove beetles on piglet cadavers at 65 forest study sites. Significant ($p < 0.05$) p-values are given in bold.

**Test**	*R statistic*	*p*	**Test**	*R statistic*	*p*
**Global for 'region'**	0.529	**0.001**	**Global for 'SMI'**	0.177	**0.001**
ALB vs. HAI	0.176	**0.002**	MEDIUM vs. HIGH	0.164	**0.04**
ALB vs. SCH	0.687	**0.001**	MEDIUM vs. LOW	0.142	**0.001**
HAI vs. SCH	0.752	**0.001**	HIGH vs. LOW	0.3	**0.003**

ALB = Schwäbische Alb, HAI = Hainich-Dün, SCH = Schorfheide-Chorin, SMI = silvicultural management intensity index.

Table S5: Results of SIMPER analysis showing those species that contributed most to the similarity of the rove beetle community composition within regions: average abundance (%) of characteristic species in each region (ALB, HAI, SCH), their contribution to the within-group similarity (%), and the cumulative total (%) of contributions (90% cut-off).

**Region ALB**<br>Average similarity: 28.59 %			
Species	Abundance	Contribution	Cumulative
T. pallipes	7.91	22.8	22.8
B. fimetarius	5.74	18.02	40.82
T. laticollis	5.78	16.47	57.29
O. tessellatus	3.17	10.66	67.96
A. mutator	3.43	9.43	77.38
O. septentrionis	1.48	6.81	84.19
M. depressus	2.61	4.22	88.41
P. decorus	1.09	2.89	91.31

**Region HAI**<br>Average similarity: 29.72 %			
Species	Abundance	Contribution	Cumulative
B. fimetarius	7.32	39.39	39.39
A. mutator	5.77	36.6	75.98
T. pallipes	7.27	7.91	83.89
P. decorus	1.14	6.47	90.37

**Region SCH**<br>Average similarity: 33.06 %			
Species	Abundance	Contribution	Cumulative
P. addendus	14.1	33.53	33.53
P. succicola	8.7	26.62	60.16
P. splendens	5.35	11.08	71.24
P. tenuicornis	5.65	7.94	79.18
O. olens	1.5	3.65	82.83
O. tessellatus	1.45	3.39	86.22
P. marginatus	3.75	2.73	88.95
O. murinus	3	2.69	91.63

ALB = Schwäbische Alb, HAI = Hainich-Dün, SCH = Schorfheide-Chorin.

Table S6: Results of SIMPER analysis showing those species that contributed most to the dissimilarity of the rove beetle community composition between regions: average abundance (%) of characteristic species for each region (ALB, HAI, SCH), their contribution to the between-group dissimilarity (%), and the cumulative total (%) of contributions (90% cut-off).

Groups ALB & HAI
Average dissimilarity = 76.39 %

Species	Group ALB Abundance	Group HAI Abundance	Contribution	Cumulative
T. pallipes	7.91	7.27	17.78	17.78
A. mutator	3.43	5.77	14.33	32.11
B. fimetarius	5.74	7.32	14.29	46.4
T. laticollis	5.78	1.18	8.87	55.28
O. tessellatus	3.17	0.95	6.03	61.31
M. depressus	2.61	0.09	4.31	65.61
P. decorus	1.09	1.14	4.15	69.76
O. septentrionis	1.48	0.32	3.52	73.29
P. addendus	0.52	0.73	2.28	75.57
A. monticola	0.61	0.55	1.9	77.47
A. crassicornis	0.26	0.32	1.17	78.64
A. castanoptera	0.39	0.18	1.04	79.67
O. tenebricosus	0.43	0	1.01	80.69
P. tenuicornis	0.26	0.23	1	81.68
O. rivulare	0	0.36	0.93	82.62
A. dadopora	0.48	0.05	0.91	83.53
A. fungi	0.43	0	0.9	84.42
P. succicola	0.3	0.18	0.85	85.27
Q. cinctus	0.57	0.09	0.84	86.11
L. lunulatus	0	0.59	0.81	86.92
P. marginatus	0.57	0	0.81	87.73
A. sodalis	0.3	0.05	0.74	88.46
A. britanniae	0.39	0	0.7	89.16
A. hansseni	0.3	0.14	0.67	89.83
P. brachypterus	0.39	0	0.66	90.49

Groups ALB & SCH
Average dissimilarity = 90.42 %

Species	Group ALB Abundance	Group SCH Abundance	Contribution	Cumulative
P. addendus	0.52	14.1	15.61	15.61
P. succicola	0.3	8.7	10.67	26.28
T. pallipes	7.91	3.1	9.2	35.49
B. fimetarius	5.74	2.25	6.85	42.33
T. laticollis	5.78	0.35	6.16	48.49
P. splendens	0.09	5.35	6.12	54.61

P. tenuicornis	0.26	5.65	5.58	60.19
A. mutator	3.43	0	4.21	64.4
O. tessellatus	3.17	1.45	3.46	67.86
P. marginatus	0.57	3.75	3.41	71.27
P. decorus	1.09	2.05	3.23	74.5
M. depressus	2.61	0.1	2.92	77.42
O. murinus	0.04	3	2.83	80.25
O. olens	0	1.5	2.39	82.63
O. septentrionis	1.48	0	2.34	84.98
P. nitidus	0	1	1.23	86.21
S. erythropterus	0	0.7	1.15	87.36
A. monticola	0.61	0.05	0.91	88.27
A. dadopora	0.48	0.15	0.69	88.96
O. tenebricosus	0.43	0	0.66	89.63
A. fungi	0.43	0	0.6	90.23

Groups HAI & SCH
Average dissimilarity = 92.11 %

Species	Group HAI Abundance	Group SCH Abundance	Contribution	Cumulative
P. addendus	0.73	14.1	16.8	16.8
P. succicola	0.18	8.7	11.59	28.39
A. mutator	5.77	0	10.67	39.06
B. fimetarius	7.32	2.25	10.66	49.72
T. pallipes	7.27	3.1	9.02	58.74
P. splendens	0	5.35	6.65	65.39
P. tenuicornis	0.23	5.65	5.93	71.32
P. decorus	1.14	2.05	3.98	75.3
P. marginatus	0	3.75	3.39	78.7
O. murinus	0	3	3.03	81.73
O. tessellatus	0.95	1.45	2.78	84.51
O. olens	0	1.5	2.62	87.13
T. laticollis	1.18	0.35	1.7	88.83
P. nitidus	0	1	1.34	90.17

ALB = Schwäbische Alb, HAI = Hainich-Dün, SCH = Schorfheide-Chorin.

Table S7: Results of SIMPER analysis showing those species that contributed most to the similarity of the rove beetle community composition within silvicultural management intensity (SMI) levels: average abundance (%) of characteristic species at each SMI level (LOW, MEDIUM, HIGH), their contribution to the within-group similarity (%), and the cumulative total (%) of contributions (90% cut-off).

SMI level 'LOW'
Average similarity: 24.59 %

Species	Abundance	Contribution	Cumulative
A. mutator	6.08	40.69	40.69
B. fimetarius	3.92	23.7	64.39
P. decorus	1.5	8.39	72.78
T. pallipes	1.73	4.47	77.25
P. addendus	2.15	4.46	81.71
A. monticola	0.46	3.11	84.82
O. tessellatus	0.92	2.55	87.37
P. succicola	2	2.52	89.89
O. septentrionis	0.69	2.5	92.39

SMI level 'MEDIUM'
Average similarity: 20.90 %

Species	Abundance	Contribution	Cumulative
T. pallipes	6.9	17.93	17.93
B. fimetarius	5.72	16.62	34.55
P. addendus	8.48	15.81	50.36
P. succicola	4.48	10.49	60.85
T. laticollis	2.72	7.51	68.36
O. tessellatus	2.66	5.52	73.88
A. mutator	1.48	4.28	78.16
P. tenuicornis	2.76	3.85	82.01
P. decorus	1.66	3.83	85.84
P. marginatus	2.69	2.23	88.07
M. depressus	1.45	2.13	90.2

SMI level 'HIGH'
Average similarity: 25.20 %

Species	Abundance	Contribution	Cumulative
T. pallipes	15.9	37.81	37.81
B. fimetarius	7	23.34	61.15
O. tessellatus	2.2	19.7	80.85
T. laticollis	6.2	11.75	92.6

Table S8: Results of SIMPER analysis showing those species that contributed most to the dissimilarity of the rove beetle community composition between silvicultural management intensity (SMI) levels: average abundance (%) of characteristic species for each SMI level (LOW, MEDIUM, HIGH), their contribution to the between-group dissimilarity (%), and the cumulative total (%) of contributions (90% cut-off).

Groups MEDIUM & HIGH
Average dissimilarity = 81.82 %

Species	Group MEDIUM Abundance	Group HIGH Abundance	Contribution	Cumulative
T. pallipes	6.9	15.9	19.72	19.72
B. fimetarius	5.72	7	11.62	31.34
P. addendus	8.48	0.8	11.08	42.42
T. laticollis	2.72	6.2	8.07	50.5
P. succicola	4.48	0.3	6.55	57.05
O. tessellatus	2.66	2.2	4.85	61.89
P. tenuicornis	2.76	0.1	2.98	64.87
P. decorus	1.66	0.4	2.82	67.69
M. depressus	1.45	0.7	2.81	70.5
A. mutator	1.48	0.5	2.8	73.29
P. marginatus	2.69	0	2.68	75.98
P. splendens	1.86	0	1.95	77.92
O. murinus	1.79	0	1.86	79.78
O. septentrionis	0.72	0.2	1.59	81.38
O. olens	0.76	0	1.39	82.77
A. monticola	0.31	0.6	1.31	84.08
S. erythropterus	0.48	0	1.02	85.1
A. crassicornis	0.28	0.2	0.99	86.08
O. tenebricosus	0.14	0.5	0.98	87.06
L. lunulatus	0.07	0.9	0.91	87.97
Q. cinctus	0.28	0.6	0.84	88.81
A. intermedia	0.07	0.2	0.78	89.59
A. dadopora	0.38	0.2	0.75	90.34

Groups MEDIUM & LOW
Average dissimilarity = 81.71 %

Species	Group MEDIUM Abundance	Group LOW Abundance	Contribution	Cumulative
P. addendus	8.48	2.15	12.24	12.24
A. mutator	1.48	6.08	10.79	23.03
B. fimetarius	5.72	3.92	10.31	33.34
T. pallipes	6.9	1.73	9.76	43.09
P. succicola	4.48	2	7.75	50.85
T. laticollis	2.72	0.96	4.48	55.33
P. tenuicornis	2.76	1.65	4.48	59.81

P. splendens	1.86	2.12	4.22	64.03
P. decorus	1.66	1.5	4.11	68.14
O. tessellatus	2.66	0.92	3.86	72
P. marginatus	2.69	0.38	2.99	74.99
M. depressus	1.45	0.58	2.83	77.82
O. murinus	1.79	0.35	2.22	80.04
O. septentrionis	0.72	0.69	2.19	82.23
O. olens	0.76	0.31	1.76	83.99
A. monticola	0.31	0.46	1.32	85.32
S. erythropterus	0.48	0	0.97	86.29
P. nitidus	0.41	0.31	0.88	87.17
A. dadopora	0.38	0.08	0.7	87.87
A. castanoptera	0.28	0.12	0.65	88.52
A. fungi	0.28	0.08	0.64	89.16
A. crassicornis	0.28	0.15	0.61	89.77
O. rivulare	0.03	0.27	0.59	90.35

Groups HIGH & LOW
Average dissimilarity = 86.41 %

Species	Group HIGH Abundance	Group LOW Abundance	Contribution	Cumulative
T. pallipes	15.9	1.73	20.77	20.77
A. mutator	0.5	6.08	14.91	35.68
B. fimetarius	7	3.92	12.23	47.91
T. laticollis	6.2	0.96	8.39	56.3
O. tessellatus	2.2	0.92	4.82	61.12
P. addendus	0.8	2.15	4.45	65.57
P. decorus	0.4	1.5	4.03	69.61
P. succicola	0.3	2	3.5	73.1
P. splendens	0	2.12	3.42	76.52
P. tenuicornis	0.1	1.65	2.53	79.04
O. septentrionis	0.2	0.69	1.86	80.91
A. monticola	0.6	0.46	1.58	82.49
M. depressus	0.7	0.58	1.38	83.87
A. crassicornis	0.2	0.15	1.13	85
O. tenebricosus	0.5	0.04	1.12	86.12
L. lunulatus	0.9	0.12	1.05	87.17
A. intermedia	0.2	0	0.93	88.1
O. rivulare	0	0.27	0.76	88.86
Q. cinctus	0.6	0.04	0.75	89.61
O. olens	0	0.31	0.65	90.26

References in supplementary file:

1. Weide, D.; Thayer, M.K.; Newton, A.F.; Betz, O. Comparative morphology of the head of selected sporophagous and non-sporophagous aleocharinae (Coleoptera: Staphylinidae): musculature and hypopharynx-prementum complex. *J. Morphol* **2010**, *271*, 910–931, doi:10.1002/jmor.10841.
2. Topp, W. Imaginal aestivation in the rove beede species *Omalium rivulare* (Coleoptera: Staphylinidae). *Entomol Gen* **1986**, *12*, 51–55.
3. Thayer, M. Staphylinidae. In *Handbook of Zoology, Vol IV Part 38 Coleoptera, Vol. 1: Morphology and Systematics (Archostemata, Adephaga, Myxophaga, Staphyliniformia, Scarabaeiformia, Elateriformia)*; Beutel, R.G., Leschen, R.A.B., Eds.; Walter de Gruyter: Berlin, Germany & New York, USA, **2005**; pp 296–344.
4. Steel, W.O. The larvae of the genera of the Omaliinae (Coleoptera: Staphylinidae) with particular reference to the British fauna. *Trans. R. Entomol. Soc. Lond.* **1970**, *122*, 1–47.
5. Lipkow, E.; Betz, O. Staphylinidae and fungi. *Faun.-Ökol.Mitt.* **2005**, *8*, 383–411.
6. Horion, A. *Faunistik der Mitteleuropäischen Käfer. Band XI: Staphylinidae. 3. Teil Habrocerinae - Aleocharinae (ohne Subtribus Athetae);* Verlagsdruckerei PH. C. W. Schmidt: Neustadt a.d. Aisch, **1967**.
7. Horion, A. *Faunistik der Mitteleuropäischen Käfer. Band X: Staphylinidae. 2. Teil Paederinae - Staphylininae;* Verlagsdruckerei PH. C. W. Schmidt: Neustadt a.d. Aisch, **1965**.
8. Horion, A. *Faunistik der Mitteleuropäischen Käfer. Band IX: Staphylinidae. 1. Teil Micropeplinae - Euaesthetinae;* Kommissionsverlag Buchdruckerei Aug. Feyel: Überlingen, Bodensee, **1963**.
9. Good, J.A.; Giller, P.S. The diet of predatory staphylinid beetles - a review of records. *Entomol.'s Mon. Mag.* **1991**, 77–89.
